# Depressive disorders among Tunisian high school teachers in the face of COVID-19

**DOI:** 10.1192/j.eurpsy.2022.1233

**Published:** 2022-09-01

**Authors:** N. Regaieg, L. Zouari, Y. Mejdoub, R. Feki, I. Gassara, N. Smaoui, S. Omri, M. Maalej, J. Ben Thabet, N. Charfi, M. Maalej

**Affiliations:** 1 Hedi Chaker University Hospital, Psychiatry C, Sfax, Tunisia; 2 Hedi Chaker University Hospital, Epidemiology, Sfax, Tunisia

**Keywords:** Depression, high school teachers, Covid-19

## Abstract

**Introduction:**

The constraints linked to COVID-19 may reduce resilience and intensify depressive feelings especially in vulnerable populations such as teachers.

**Objectives:**

To understand the psychological distress in terms of depression of the COVID-19 pandemic among secondary school teachers in Sfax, Tunisia and to determine the correlated factors.

**Methods:**

This was a descriptive and analytical cross-sectional study of 97 medium and high school teachers from Sfax, Tunisia. The study was conducted on google drive in May 2021, including an information sheet and the Patient Health Questionnaire (PHQ)-9 to assess depression.

**Results:**

In our study, the sex-ratio (M/F) was 0.32 and the average age was 44.23 years old. The median professional seniority was 16 years (minimum=1, maximum=37). Suicidal thoughts were described by 10.4% of teachers while 54.2% reported the presence of sleep disturbances since the onset of the pandemic. The median PHQ-9 score was 8 (Q1=4, Q3=15). The distribution of scores indicated that 59.7% of participants had no to mild symptoms of depression while 40.3% had moderate to severe depression. Furthermore, the presence of depression was associated with an age ≤ 40 years old (p=0.037), a professional seniority ≤ 20 years (p=0.035), the female gender (p=0.005), the presence of sleep disturbances (p<0.001) as well as with suicidal thoughts (p=0.006).

**Conclusions:**

It seems that COVID-19 health situation in education have led to the emergence of a teacher overexertion and a depth adaptation to the new environment demands. Thus, clinical attention to the depression level of the teachers is certainly warranted.

**Disclosure:**

No significant relationships.

